# Endothelial activation and fibrotic changes are impeded by laminar flow-induced CHK1-SENP2 activity through mechanisms distinct from endothelial-to-mesenchymal cell transition

**DOI:** 10.3389/fcvm.2023.1187490

**Published:** 2023-08-30

**Authors:** Minh T. H. Nguyen, Masaki Imanishi, Shengyu Li, Khanh Chau, Priyanka Banerjee, Loka reddy Velatooru, Kyung Ae Ko, Venkata S. K. Samanthapudi, Young J. Gi, Ling-Ling Lee, Rei J. Abe, Elena McBeath, Anita Deswal, Steven H. Lin, Nicolas L. Palaskas, Robert Dantzer, Keigi Fujiwara, Mae K. Borchrdt, Estefani Berrios Turcios, Elizabeth A. Olmsted-Davis, Sivareddy Kotla, John P. Cooke, Guangyu Wang, Jun-ichi Abe, Nhat-Tu Le

**Affiliations:** ^1^ Center for Cardiovascular Regeneration, Department of Cardiovascular Sciences, Houston Methodist Research Institute, Houston, TX, United States; ^2^ Department of Life Science, Vietnam Academy of Science and Technology, University of Science and Technology of Hanoi, Hanoi, Vietnam; ^3^ Department of Cardiology, The University of Texas MD Anderson Cancer Center, Houston, TX, United States; ^4^ Department of Symptom Research, The University of Texas MD Anderson Cancer Center, Houston, TX, United States

**Keywords:** atherosclerosis, endothelial activation, laminar flow, CHK1, SENP2, SUMOylation, fibrotic changes

## Abstract

**Background:**

The deSUMOylase sentrin-specific isopeptidase 2 (SENP2) plays a crucial role in atheroprotection. However, the phosphorylation of SENP2 at T368 under disturbed flow (D-flow) conditions hinders its nuclear function and promotes endothelial cell (EC) activation. SUMOylation has been implicated in D-flow-induced endothelial-to-mesenchymal transition (endoMT), but the precise role of SENP2 in counteracting this process remains unclear.

**Method:**

We developed a phospho-specific SENP2 S344 antibody and generated knock-in (KI) mice with a phospho-site mutation of SENP2 S344A using CRISPR/Cas9 technology. We then investigated the effects of SENP2 S344 phosphorylation under two distinct flow patterns and during hypercholesteremia (HC)-mediated EC activation.

**Result:**

Our findings demonstrate that laminar flow (L-flow) induces phosphorylation of SENP2 at S344 through the activation of checkpoint kinase 1 (CHK1), leading to the inhibition of ERK5 and p53 SUMOylation and subsequent suppression of EC activation. We observed a significant increase in lipid-laden lesions in both the aortic arch (under D-flow) and descending aorta (under L-flow) of female hypercholesterolemic SENP2 S344A KI mice. In male hypercholesterolemic SENP2 S344A KI mice, larger lipid-laden lesions were only observed in the aortic arch area, suggesting a weaker HC-mediated atherogenesis in male mice compared to females. Ionizing radiation (IR) reduced CHK1 expression and SENP2 S344 phosphorylation, attenuating the pro-atherosclerotic effects observed in female SENP2 S344A KI mice after bone marrow transplantation (BMT), particularly in L-flow areas. The phospho-site mutation SENP2 S344A upregulates processes associated with EC activation, including inflammation, migration, and proliferation. Additionally, fibrotic changes and up-regulated expression of EC marker genes were observed. Apoptosis was augmented in ECs derived from the lungs of SENP2 S344A KI mice, primarily through the inhibition of ERK5-mediated expression of DNA damage-induced apoptosis suppressor (DDIAS).

**Summary:**

In this study, we have revealed a novel mechanism underlying the suppressive effects of L-flow on EC inflammation, migration, proliferation, apoptosis, and fibrotic changes through promoting CHK1-induced SENP2 S344 phosphorylation. The phospho-site mutation SENP2 S344A responds to L-flow through a distinct mechanism, which involves the upregulation of both mesenchymal and EC marker genes.

## Introduction

Blood flow patterns have a significant impact on disease progression *in vivo,* but the underlying biological mechanisms are not fully understood*.* It has been observed that exposure to D-flow, but not L-flow (1, 2), in vascular regions of ECs contributes to the development of atherosclerosis (2). The deSUMOylation enzyme SENP2, containing nuclear localization and export signals with nucleocytoplasmic shuttling activity, plays a crucial role in regulating the SUMOylation of various proteins involved in important cellular processes. These proteins include extracellular signal-regulated kinase 5 (ERK5), tumor suppressor protein p53, focal adhesion kinase (FAK), and membrane associated guanylate kinase WW and PDZ domain containing 1 (MAGI1) (3–9). Our previously studies have demonstrated that D-flow activates a redox sensitive ribosomal S6 kinase p90RSK, leading to phosphorylation of SENP2 at T368. This phosphorylation event subsequently triggers the SUMOylation of ERK5 and p53, promoting EC inflammation and apoptosis. Consequently, EC dysfunction ensues, contributing to the development of atherosclerosis (3–5, 9–12). In contrast, we have also observed that L-flow inhibits the SUMOylation of ERK5 and p53 without affecting SENP2 T368 phosphorylation (2–4, 14). This suggests that the atheroprotective effects of L-flow are independent of SENP2 T368 phosphorylation.

Atherogenesis is closely associated with DNA damage in cells of the vessel walls, leading to the activation of DNA damage response (DDR) pathways (14–16). DDR plays a crucial role in maintaining the genetic stability and integrity of cells exposed to DNA-damaging agents such as radiotherapy and chemotherapy, commonly used in cancer treatment (17). DDR achieves this by initiating cell cycle arrest for DNA repair and promoting cell apoptosis and senescence to prevent the propagation of damaged DNA. The main DDR pathways are governed by the ataxia-telangiectasia-mutated (ATM) and ataxia-telangiectasia and Rad3-related (ATR) kinases, which phosphorylate proteins at sites of DNA damage. For instance, phosphorylation of the Serine 139 residue of the histone variant H2AX leads to the formation of γH2AX and activates check point kinase (CHK) (18). CHK1 is activated by ATR and plays a critical role in regulating DDR to prevent the propagation of DNA damage. It induces cell cycle arrest, senescence, and apoptosis, thereby halting the progression of cell with damaged DNA (18, 19). CHK1 activation is particularly important in preventing cells with defective G1 checkpoint, often observed due to p53 mutations, from entering mitosis. Moreover, CHK1 also suppresses replicative stress by inhibiting excess origin firing, especially in cells with activated oncogenes.

EC activation is a significant contributor to atherosclerosis. Various mechanisms contribute to EC activation, including the generation of adhesion molecules and chemokines (inflammation), migration, proliferation, and apoptosis. L-flow inhibits these processes, while D-flow promotes them (20). ECs can undergo transdifferentiation to a mesenchymal phenotype through a process called endothelial-to-mesenchymal transition (endoMT) (21, 22). During endoMT, the expression of EC-specific genes such as cluster of differentiation 31 [CD31 or platelet endothelial cells adhesion molecule 1 (PECAM1)], vascular endothelial cadherin (VE-Cadherin or CDH5), von Willebrand factor (vWF), tyrosine kinase with immunoglobulin-like and EGF-like domains 1 (TIE1), and TEK receptor tyrosine kinase (TIE2) is downregulated (21, 23, 24) while the expression of mesenchymal cell-specific genes such as α-smooth muscle actin (αSMA), extra domain A (EDA) of fibronectin, N-cadherin, vimentin, fibroblast specific protein 1 (FSP1), fibroblast activating protein (FAP), and Twist-related protein 1 (TWIST1) is upregulated (23, 24). Although endoMT was initially discovered during embryonic cardiac development, recent studies have suggested its involvement in atherosclerosis (25–30). Importantly, blood flow plays a critical role in regulating endoMT. Moonen and colleagues have shown that L-flow-induced activation of ERK5 can suppress endoMT (31). It has become evident that the two different flow patterns, L-flow and D-flow, regulate EC activation and endoMT differently but in concert. While several studies have focused on how flow regulates individual biological responses, to the best of our knowledge, it remains unclear how flow regulates all the processes associated with EC activation and endoMT and their contribution to atherosclerosis.

Radiation therapy-induced cardiovascular disease is a significant cause of morbidity and mortality among cancer survivors, particularly in breast cancer (32), lymphoma (33, 34), and lung cancer (35, 36). A study by Darby and colleagues revealed that there is an increase of 7.4% in major coronary events per gray (Gy) of mean heart dose, and within the first 4 years of radiation therapy, there is an increase of 16.3% per Gy of mean heart dose. Even small increments in mean heart dose are associated with an elevated risk of major coronary events (2 Gy: 10%; 2–4 Gy: 30%; 5–9 Gy: 40%; ≥10 Gy: 116%) compared to patients receiving no heart dose (37). Radiation therapy leads to acute macro- and micro-vascular injury by causing EC injury and activating cardiac monocyte and macrophage. However, the regulatory mechanisms underlying these effects remain unclear, and there are currently no effective treatment available to prevent radiation-induced cardiovascular disease.

In our research, we explored publicly available datasets to investigate other phosphorylation sites of SENP2, as the atheroprotective effect of L-flow seemed independent of SENP2 T368 phosphorylation. Our analysis revealed that SENP2 can undergo phosphorylation at S344 (38, 39) by CHK1, a kinase identified through chemical and genetic approaches combined with high-resolution mass-spectrometry (19, 38, 39). Additionally, we found that exposure to L-flow enhances the phosphorylation of CHK1 at S280 and SENP2 at S344 in ECs. Intriguingly, we observed that radiation disrupts this pathway by reducing CHK1 expression. However, the functional role, regulatory mechanism, and contribution of CHK1-mediated SENP2 S344 phosphorylation to EC activation, endoMT, and atherogenesis are still poorly understood. Therefore, our study aims to investigate the functional role and regulatory mechanism of CHK1-mediated SENP2 S344 phosphorylation induced by L-flow and its subsequent impact on atherogenesis.

## Methods

The data supporting the findings of this study are available from the corresponding authors upon reasonable request.

### Plasmids, adeno-associated virus (AAV), and adenoviruses

The plasmid containing human SENP2 WT was obtained from Addgene (#18047) (40). To generate the phospho-site mutation SENP2 S344A, a Quik Change site-directed mutagenesis kit (Agilent Technologies) was used following the manufacturer's instructions. Adenoviruses expressing human SENP2 WT and S344A (Ad SENP2 WT, Ad SENP2 S344A) were generated by cloning each corresponding insert from the pCMV vector into the pENTR1A vector (Life Technologies, #A10462) at sites recognized by the restriction enzymes EcoRI and SalI. A recombinase reaction was performed to obtain a pDEST-based vector using the Invitrogen Gateway LR Clonase II Enzyme mix (#11791100) according to the manufacturer's instructions. For control purposes, adenovirus containing b-galactosidase (Ad-LacZ) was used (9). Plasmids containing human DDIAS (C11orf82, #RC206347) and pCMV6-Entry with C-terminal Myc-DDK Tag (#PS100001) were purchased from OriGene Technologies, Inc. Additionally, a plasmid encoding mPCSK9 was obtained from Addgene (#58376) (41). A recombinant adeno-associated virus serotype 8 (rAAV8) expressing mPCSK9 under the control of the liver-specific promoter controlling region-apolipoprotein enhancer/alpha1-antitrypsin (rAAV8-HCRApoE/hAAT) was produced by the University of North Carolina Vector Core (Chapel Hill, NC).

### Antibodies and reagents

Phospho-specific SENP2 S344 antibody was custom produced by Pierce Biotechnology Inc. Rabbits were immunized with a synthesized peptide corresponding to amino acids 334–354 of the human SENP2 sequence (Ac-GSNGLLRRKVS*IIETKEKNCS). Adverse reactions, including lesion formation, loss of appetite, and non-responsiveness, were monitored during the immunization process. Once the rabbits passed the initial evaluation, the remaining immunization protocol was carried out according to the manufacturer's protocol. After the completion of the immunization process, the rabbits were euthanized, and the obtained sera were affinity purified. The following antibodies were purchased from Cell Signaling Technology (Beverly, MA, USA): ERK5 (#3372), cleaved caspase 3 (#9661), ICAM1 (#4915, #67836), p53 (#9282), VCAM1 (#13662), phospho-CHK1 S280 (#2347S), phospho-CHK1 S345 (#2348S), and phospho-CHK1 S317 (#12302S). The following antibodies were purchased from Abcam (Waltham, MA, USA) SENP2 (#58418), VCAM1 (#13407), α-SMA (#ab5694). CHK1 (sc-8408) was purchased from Santa Cruz biotechnology (Dallas, TX). SENP2 antibody (NBP1-31217) and β-actin antibody (NB600-532) were purchased from Novus Biologicals (Briarwood Ave, CO, USA). The following antibodies and reagents were purchased from Sigma-Aldrich (St. Louis, MO, USA): DDIAS (HPA038541), TWIST1 (ABD29), protease inhibitor cocktail (P8340), PMSF (#36978), NEM (E3876), and diphenyleneiodonium chloride (D2926). SUMO2/3 antibody (AP1224a) was purchased from Abcepta, Inc (San Diego, CA). The Quick ChangeII Site-Directed Mutagenesis Kit (#200523) was purchased from Agilent Technology (Santa Clara, CA, USA). Lipofectamine 2,000 transfection reagent (#11668027) was purchased from Thermo Fisher Scientific, Waltham, MA). CHK1 inhibitor (GDC 0575) and ERK5 inhibitor (XMD8-92, #S7525) were purchased from Selleck Chemicals LLC (Houston, TX, USA), dissolved in DMSO, and pretreated to ECs prior to flow exposure at the doses indicated in the figures.

### Animal studies

Procedures involving mice conducted in accordance with the guidelines and regulations set forth by the Institutional Care and Use Committees (IACUC) of the Texas A&M Institute of Biosciences and Technology (2014-0231, 2017-0154) and the University of Texas MD Anderson Cancer Center (UTMDACC; 00001652, 00001109), adhering to the NIH standards outlined in the Guide for the Care and Use of Laboratory Animals (DHHS pub. NIH 85-23 Rev.1985). The mice were provided with an appropriate irradiated diet and ultra-filtered water. They were housed in a facility at UTMDACC, which is AAALAC certified and maintained with an ambient temperature of 22°C with a 12 h light/12 h dark cycle (42). The mice were housed in a cage-level barrier system with heat-treated wood chip bedding and enrichment material (nestlets). Cages and water were changed on a weekly basis. To study the mouse counterpart of human SENP2 S344 (S343, referred to as “mouse SENP2 S344” for consistency), we generated SENP2 S344A knock-in (KI) mice using CRISPR/Cas9 technology. The gRNAs for targeting SENP2 S344 were selected based on minimal off-target cuts using an off-target program (http://crispr.mit.edu/), and their *in vivo* cutting efficiency was assessed using an efficiency-of-cutting program (http://crispr.dfci.harvard.edu/SSC/). Three gRNAs that cut closest to the S344 codon while meeting the off-target and efficiency criteria were chosen for further testing. The gRNAs, along with an unrelated gRNA, were incubated with an SENP2 plasmid and Cas9 following the instructions provided with the Clontech Cas9 guide-it Screening Kit. Gel electrophoresis confirmed 100% accuracy of the three SENP2 S344 gRNAs in cutting target sequence, while the control gRNA did not show any cutting activity (data not shown). The 110 bp donor DNAs containing the S344A mutation, along with a silent mutation in the PAM sequence (resulting in the same amino acid but with a different codon), were synthesized by Sigma-Aldrich. Excess single-strand donor DNAs were used for asymmetric PCR and PCR clean-up. The final product was then sent to the Genetically Engineered Mouse Facility at UTMDACC. Each donor DNA, along with its corresponding gRNA and Cas9, was microinjected into the pronucleus of fertilized C57/BL6 mouse eggs and implanted into recipient mice.

To measure the levels of HDL and LDL cholesterol, a cholesterol assay kit for mice (#EHDL-100, Bioassay System, Hayward, CA) was used (43).

### Tissue preparation, histologic evaluation, and quantification of lesion size

We administered a single dose of rAAV8-mPCSK9 (1 × 10^11^/mouse) via tail veins of eight-week-old mice, which were then fed a high-fat diet (HFD) consisting of 21% fat, 0.15% cholesterol, 19.5% casein (#TD.88137; Envigo, NJ) (44) for 16 weeks. Mouse body weight was recorded before and after HFD. At the end of the experiment, mice were euthanized using CO_2_ inhalation, and blood samples were collected for LDL cholesterol assay (45). The arterial tree was perfused through the left ventricle with saline containing heparin (40 USP U/ml), followed by 10% neutral-buffered formalin in PBS (10 min). The aorta was dissected from the heart to the iliac bifurcation and opened along the ventral midline. En face prepared aortas were washed in PBS, dipped in 60% isopropyl alcohol, and stained with 0.3% Oil Red O (ORO) in 60% isopropyl alcohol for 30 min. Images were captured using a digital camera mounted on a Nikon SMZ1000 stereomicroscope, and image analysis was performed using the ImageJ software (5, 46) in a double-blinded manner.

### Bone marrow transplantation (BMT)

To perform BMT, lethally irradiated bone marrow cells obtained from eight-to-nine-week-old SENP2 S344A KI or WT mice were replaced with bone marrow cells obtained from WT mice (47). Following a recovery period of 6 weeks, the recipient mice were administered a single dose of rAAV8-mPCSK9 and were fed a HFD for 22 weeks (45). To confirm the successful completion of the BMT, PCR was conducted on genomic DNA extracted from peripheral blood of the recipient mice, using specific primers for WT (forward: CAC GTA TTC ACT ACC CAA TGT GGA GTT C; reverse: AAG TTC TTT TCC TTT ATC TCA AGC ACT GA) and SENP2 S344A KI (forward: CAC GTA TTC ACT ACC CAA TGT GGA GTT C, reverse: GTT CTT TTC CTT AAT CTC AAG CAC TGC). Lipid-laden lesions in the mouse aortas were identified through Oil Red O staining of *en-face* preparations. The aortic valve leaflets, sectioned at the center area between the free edge and attachment site at the annulus, were stained using Trichrome Stain Kit (ab150686; abcam). Histological evaluation was performed to assess the changes in the aortic valves of the BMT recipients.

### Isolation and culture of human umbilical vein ECs (HUVECs)

HUVECs were isolated by performing a collagenase digestion of the endothelium obtained from human umbilical cord veins. Subsequently, we cultured the cells on dishes or flasks that were coated with 0.2% gelatin type A (#901771; MP Biomedicals, Santa Ana, CA, USA) in EC medium [ECM, #1001, Science Cell, San Diego, CA, USA]. The study received approval from the Institutional Review Board (IRB Pro00020559) at the Houston Methodist Research Institute (HMRI) and UTMDACC (IRB RM00000535-RN01). Informed consent was not required for this particular study.

### Isolation and culture of mouse lung ECs

The isolation of ECs from mouse lungs was approved by the IACUC at the HMRI (IS00006725), and the procedure followed previously described method (16, 21, 22). Lungs from mice aged six to eight weeks were carefully washed with cold PBS, finely minced using scissors, and then digested using collagenase. To capture the ECs from the mouse lungs, we utilized sheep anti-rat PECAM-1-conjugated Dynabeads (#11035, Invitrogen, Carlsbad, CA, USA). Subsequently, the captured cells were cultured in DMEM (#SH30243.0, Hyclone, Logan, UT, USA) supplemented with 10% FBS (#F2442, Sigma-Aldrich, Saint Louis, MO), 1% ECGS (Promo Cell, Heidelberg, Germany), 25 mM HEPES (#25-060-CI, Corning, Manassas, VA, USA), 1% non-essential amino acid solution (#25-025-CI, Corning, Manassas, VA, USA), 100 mg/ml heparin (#67457037399, Mylan Institutional, Rockford, IL, USA) and 1% penicillin/streptomycin (#30-002-CI, Corning, Manassas, VA, USA).

### siRNA and plasmid transfection

To degrade human CHK1, we utilized siRNA targeting nucleotides in the coding sequence 281–301 (AAGCGUGCCGUAGACUGUCCA), along with non-target control sequences, which were purchased from Sigma-Aldrich (Burlington, MA, USA). The siRNA and plasmid transfection procedure was conducted following our previously described methods (5, 44). GIBCO Opti-MEM reduced serum medium (#31985070; Thermo Fisher Scientific) supplemented with Plus (#11514015) and Lipofectamine (#18324020) obtained from Life Technologies were used during transfection. Following transfection, the cells were allowed to recover for 24–48 h before further processing.

### RNA-sequencing-based genome wide gene expression study

Total RNA was extracted from ECs isolated from the lungs of SENP2 S344A KI and WT mice following 24 h of L-flow exposure. The RNeasy Plus Micro Kit (#74034, QIAGEN, Germ: antown, MD) was used for RNA extraction. Subsequently, we shipped the RNA samples to Beijing Genomic Institution (BGI, Shenzhen, China) for mRNA preparation, library construction, and sequencing using the BGISEQ-500 platform. The clean tags obtained from sequencing were mapped to the reference genome and genes available at the Mice Genome Annotation Project 2, allowing for up to one mismatch. The original sequencing data have been deposited in the Gene Expression Omnibus (GEO) databse with the accession number GSE222511 and token snyjiikwrdwntcz. Aligment of the paired-end RNA-seq reads to the mouse genome (gencode.vM27) was performed using Kallisto (v0.46.0) with default parameters. DESeq2 (v2.0.12) was used to calculate gene expression levels and identify differently expressed genes (DEGs). Gene expression was measured in transcripts per million (TPM), and DEGs were defined based on *Q* value ≤0.05 as a threshold. Pathway enrichment analysis was conducted using DAVID (https://david-d.ncifcrf.gov) by calculating *p*-values for each Gene Ontology (GO) term with a modified Fisher's exact test. Significantly enriched GO terms were selected using a *Q* value <0.05 as the threshold, and a Venn diagram was constructed to identify co-expressed DEGs among the samples using R (v3.5.1). The transcriptional profiles of the KI and WT groups were analyzed, and heatmaps were generated using MORPHEUS https://software.broadinstitute.org/morpheus/.

### Immunoblotting and SUMOylation

Protein extraction was performed using radioimmunoprecipitation assay (RIPA) buffer (Tris-HCl pH 7.4 50 mM, NaCl 150 mM, ethylenediaminetetraacetic acid 1 mM, Nonidet P-40 1%, sodium dodecyl sulfate 0.1%, sodium deoxycholate 0.25%) or obtained from EMD Millipore (#20-118, Billerica MA, USA) suplemented with mammalian protease inhibitor cocktail (#p8340; Sigma-Roche, Mannheim, Germany), phenylmethylsulfonyl fluoride 1 mM (#36978; Thermo Fisher Scientific), and N-ethylmaleimide 10 mM (#E3876; Sigma-Roche, Mannheim, Germany) (5). Lysates were centrifuged (15,000 rpm/20 min/4°C) to remove debris, and protein concentration was determined using DC™ Protein Assay Kit I (#5000111; Bio-Rad, Hercules CA, USA). Equal amounts of protein were loaded onto sodium dodecyl sulfate (SDS)-polyacrylamide gel electrophoresis (SDS-PAGE) gels and then transferred onto Immobilon-P transfer membranes (#IPVH00010; Merc Millipore, Tullagreen, IRL). The membranes were incubated in 3% BSA/TBST solution (10 mM Tris-HCl, 0.15 M NaCl, 0.1% Tween 20, pH 8.0) at room temperature (1 h), washed in TBST, and then incubated with specific antibodies (500–1,000 dilution) with mild agitation overnight at 4°C. After washing three times (10 min each), the membranes were incubated with HRP-conjugated secondary antibodies (4,500–5,500 dilution), washed again, and chemiluminescence was detected using an ECL substrate (NEL105001EA; Perkin Elmer. Inc, Waltham, MA, USA). Signal intensities from the immunoblotted membranes were quantified using ImageJ. For SUMOylation, lysates were immunoprecipitated with an antibody that specifically recognizes SUMO2/3 (Signal-Seeker^TM^ SUMOylation 2/3 Detection Kit #BK162; Cytoskeleton, Inc.) and then immunoblotted with ERK5 or p53 antibody to detect SUMOylated ERK5 or p53. The same pulldown samples were also immunoblotted with a SUMO2/3 antibody to confirm equal amounts of SUMO2/3 were pulled down. A control bead was used as a reference.

### Automated capillary electrophoresis Western analysis (Wes; proteinSimple, San Jose, Ca, USA)

Lysates were mixed with a 5X fluorescent master mix containing 200 mM DTT, heated at 95°C for 5 min, and 5 μl of 0.4–1 mg/ml protein was loaded onto a Wes plate (#004-600) using a 12–230 kDa Separation Module (#SM-W003) with either a rabbit (#DM-001) or mouse (#DM-002) detection module. Lysates, blocking buffer, primary antibodies, HRP-conjugated secondary antibodies, and luminol-peroxide were dispensed onto the Wes plate. β-actin antibody was used as a loading control and was multiplexed with the primary antibodies for all samples. Capillary electrophoresis was performed using the instrument's default settings: separation time of 25 min, separation voltage of 375 V, blocking for 5 min, and primary and secondary antibodies incubation for 30 min. Automatically detected standards and peaks were manually inspected, and the data were analyzed using the built-in Compass software (ProteinSimple) (48, 49).

### Cone-and-plate devices for studying different flow patterns *in vitro*

In our study, we utilized cone-and-plate devices to investigate the impact of various flow patterns on HUVECs *in vitro* (50). The cone-and-plate method's principle has been described in detail elsewhere (51). To generate D-flow, we used cones with 1-mm deep radial grooves, which induced cobble stone-like cell shapes, mimicking the *in vivo* d-flow conditions (50). It is important to note that D-flow exhibits a turbulent flow pattern, making shear stress calculations unfeasible, unlike oscillatory flow. For L-flow experiments, we utilized flat cones (smooth cones) and observed that HUVECs assumed elongated shapes, resembling the condition observed under L-flow *in vivo* (50). Furthermore, we confirmed the successful generation of L-flow by detecting the phosphorylation of ERK5 at T-E-Y residues (50, 52). As L-flow inhibits EC inflammation while d-flow promotes it, interpreting an increase in EC inflammatory gene expression becomes challenging. It remains unclear whether such increase is due to the heightened proinflammatory effects of D-flow or the diminished anti-inflammatory effects of L-flow. To isolate the effect of each flow pattern, we included a control group with no flow (static conditions).

### *In vitro* EC migration assay

Sub-confluent HUVECs were transduced with Ad-SENP2 WT or S344A. The following day, a vertical region at the center of the HUVEC layer was scratched using a sterile 200 μl pipette tip. The culture media was then aspirated, and the cells were washed three times in PBS to remove any detached cells. Fresh ECM was added to the wells. After 6 h of L-flow exposure, the cells were photographed under a microscope, and the images were analyzed using Image J software. The cell migration rate was calculated as follows: length of initial cell-free vertical scratch − length of remaining cell-free region after L-flow) / length of initial cell-free vertical scratch × 100%.

### Statistical analysis

To assess the statistical significance of the differences between experimental groups, we initially conducted a Shapiro-Wilk test to evaluate the normality of each group. For normality distributed data, we performed an ordinary one-way ANOVA followed by Fisher's LSD test for multiple group comparisons or an unpaired Student *t*-test for two group comparisons. In the case of 2-by-2 experiments, we employed a two-way ANOVA analysis instead of a one-way ANOVA. If the data did not meet the assumptions of normality, we employed a Brown-Forsythe and Welch ANOVA or unpaired *t*-test with Welch's correction using Prism software (GraphPad Software). We considered *p*-values less than 0.05 as statistically significant.

### Data availability

The RNA-sequencing data generated in this study have been deposited into the NCBI's Gene Expression Omnibus database under accession GSE95066. The sequence of SENP2 has been deposited in GenBank under accession KY651081. All other data, analytic methods, and study materials that support the findings of this study are included in the Data supplement or can be obtained from the corresponding authors upon reasonable request.

## Results

### CHK1 regulates L-flow-induced phosphorylation of SENP2 at S344 residue, subsequently suppressesing the SUMOylation of ERK5 and p53

**Figure 1:**
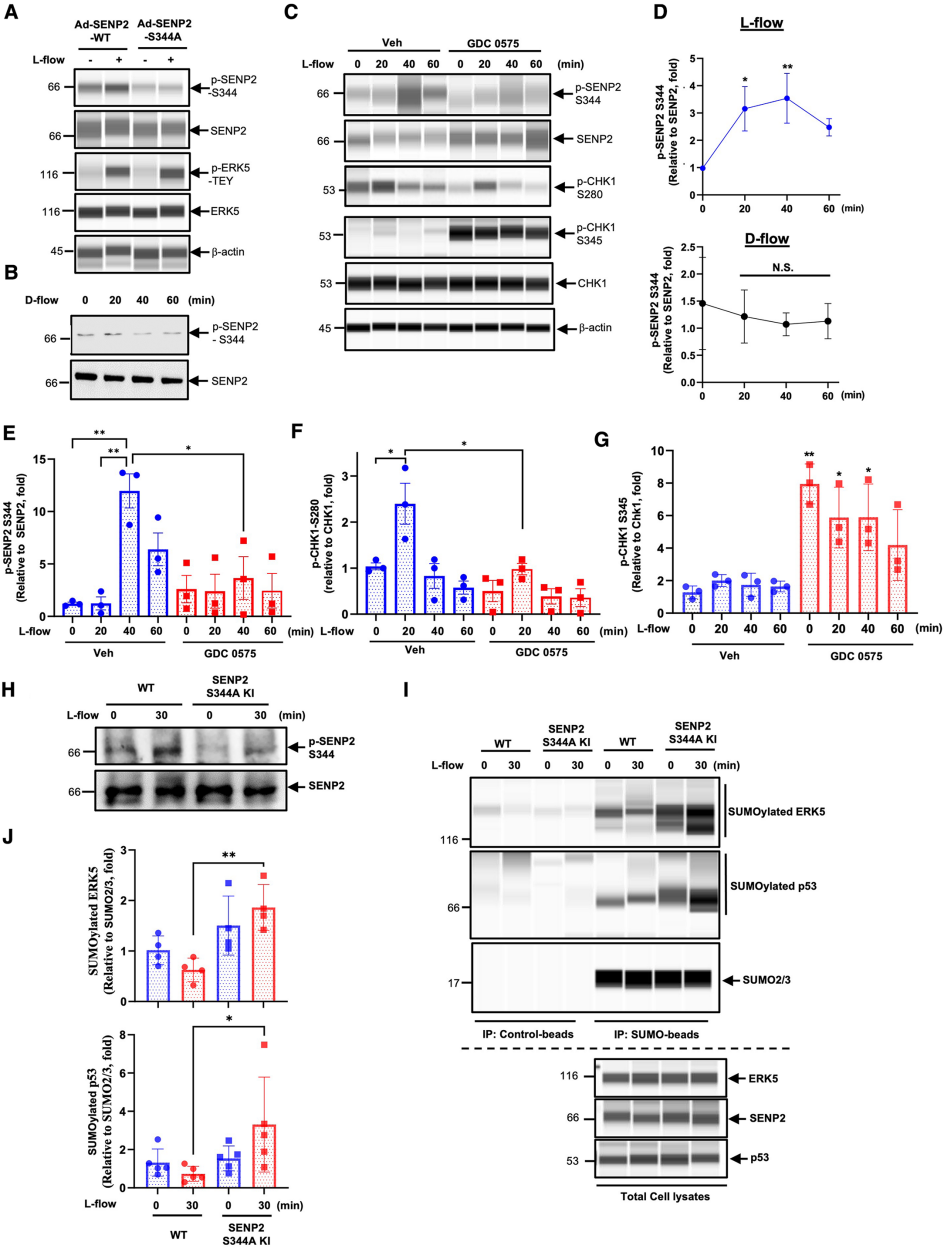
CHK1 regulates L-flow-induced SENP2 S344 phosphorylation, subsequently suppresses ERK5 and p53 SUMOylation: (**A**) HUVECs transduced with Ad-SENP2 WT or Ad-SENP2 S344A were exposed to L-flow for 0 and 30 min, and the levels of SENP2 (p-S344 and total), ERK5 (p-T-E-Y and total), and actin (loading control) were determined using Wes. An increase in p-ERK5-T-E-Y indicated successful generation of L-flow, in addition to elongated cell shape (50, 52). (**B**) HUVECs were exposed to D-flow for 0, 20, 40, and 60 min, and the levels of SENP2 (p-S344 and total) were determined using immunoblotting. (**C**) HUVECs pretreated with 250 nM GDC 0575 were exposed to L-flow for 0, 20, 40, and 60 min, and the levels of SENP2 (p-S344 and total), CHK1 (p-S280, p-S345, and total), and actin were determined using Wes. (**D**, upper panel) The graphs present quantified data from 3 independent experiments from (**C**) (*n* = 3) (*p < 0.05, **p < 0.01, two-way ANOVA). **(D**, lower panel**)** The graphs present quantified data from 3 independent experiments from (**B**) (*n* = 3). N.S. indicates no significance. **(E–G)** The graphs present quantified data from 3 independent experiments from (**C**) (*n* = 3) (*p < 0.05, **p < 0.01, two-way ANOVA). **(H)** WT mouse lung ECs (WT MLECs) and SENP2 S344A KI MLECs were exposed to L-flow for 0 and 30 min, and the levels of SENP2 (p-S344 and total) were determined by immunoblotting. **(I**) WT MLECs and SENP2 S344A KI MLECs were exposed to L-flow for 0 and 30 min. Lysates were immuno-precipitated with the SUMO2/3 antibody (Signal-Seeker^TM^ SUMOylation 2/3 Detection Kit, #BK162; Cytoskeleton, Inc.), and ERK5 or p53 antibody was used to detect SUMOylated ERK5 or p53 using Wes, respectively. The SUMO2/3 antibody was also used to confirm equal immunoprecipitation across the samples. Control beads were used as the negative control. ERK5, SENP2, and p53antibodies were used to detect their expression in total cell lysates using Wes. (**J**) The graphs present quantified data from 4 (upper panel) and 5 (lower panel) independent experiments (*p < 0.05, two-way ANOVA).

Based on publicly available mass spectrometry-based phospho-proteomic datasets that revealed phosphorylation of SENP2 at the Serine344 (S344) residue (38, 39), we aimed to investigate if this phosphorylation occurs in ECs in response to L-flow. To address this, we generated a phospho-specific SENP2 S344 antibody and transduced HUVECs with adenovirus expressing either the phospho-site mutation SENP2 S344A or the wild-type form (Ad-SENP2 S344A or Ad-SENP2 WT). Subsequently, these HUVECs were exposed to L-flow. Following L-flow, we observed an increase in SENP2 S344 phosphorylation, which was effectively abolished by transduction with Ad-SENP2 S344A (Figures 1A,C,D). By contrast, there was no significant increase in SENP2 S344 phosphorylation in response to D-flow (Figures 1B,D).

Another publicly available mass spectrometry-based dataset revealed that CHK1 phosphorylates SENP2 at the S344 residue. However, the functional consequence of this phosphorylation has not been characterized yet (19). To investigate the involvement of CHK1 in L-flow-mediated SENP2 S344 phosphorylation, we pre-treated HUVECs with the CHK1 inhibitor, GDC 0575 (53), prior to L-flow exposure. Remarkably, the pretreatment with GDC 0575 resulted in a significant decrease in SENP2 S344 phosphorylation (Figures 1C,E). These findings suggest that CHK1 plays a role in L-flow-mediated SENP2 S344 phosphorylation.

Moreover, since CHK1 activation is known to be regulated by the CHK1-mediated phosphatase 2A circuit (54), we assessed the effect of GDC 0575 on CHK1 activation. Interestingly, treatment with GDC 0575 led to an increase in CHK1 S345 phosphorylation, indicates effective suppression of CHK1 activation due to the inhibition of phosphatase 2A activity (Figures 1C,G). Notably, we observed an increase in CHK1 phosphorylation at S280, but not S345, in the vehicle-treated HUVECs after L-flow, which was abolished by GDC 0575 (Figures 1C,F,G). It is worth noting that phosphorylation of CHK1 at S280, but not S345, promotes CHK1 nuclear translocation (45, 55, 56). Therefore, it is possible that CHK1-mediated SENP2 S344 phosphorylation following L-flow is regulated by CHK1 S280 phosphorylation-driven CHK1 nuclear translocation rather than CHK1 S345 phosphorylation-dependent kinase activation.

To investigate the functions and regulatory mechanisms of SENP2 S344 phosphorylation, we utilized CRISPR/Cas9 technology to generate mice with a phospho-site mutation in SENP2 at S344 (SENP2 S344A KI mice) (47). We isolated ECs from the lungs of the SENP2 S344A KI mice (SENP2 S344A KI MLECs) and examined the effects of L-flow on SENP2 S344 phosphorylation. Notably, we observed that L-flow-induced SENP2 S344 phosphorylation was significantly inhibited in SENP2 S344A KI MLECs (Figure 1H). Furthermore, we investigated the impact of SENP2 S344 phosphorylation on the SUMOylation of ERK5 and p53. Interestingly, we observed an increase in the SUMOylation of ERK5 and p53 in SENP2 S344A KI MLECs specifically after L-flow stimulation, while no significant changes were observed under static conditions, compared to those in WT MLECs (Figures 1I,J). These findings strongly suggest that SENP2 S344 phosphorylation induced by L-flow plays a crucial role in inhibiting the SUMOylation of ERK5 and p53 through modulating SENP2 deSUMOylation activity.

### SENP2 S344 phosphorylation suppresses EC inflammation and apoptosis *in vivo*

**Figure 2:**
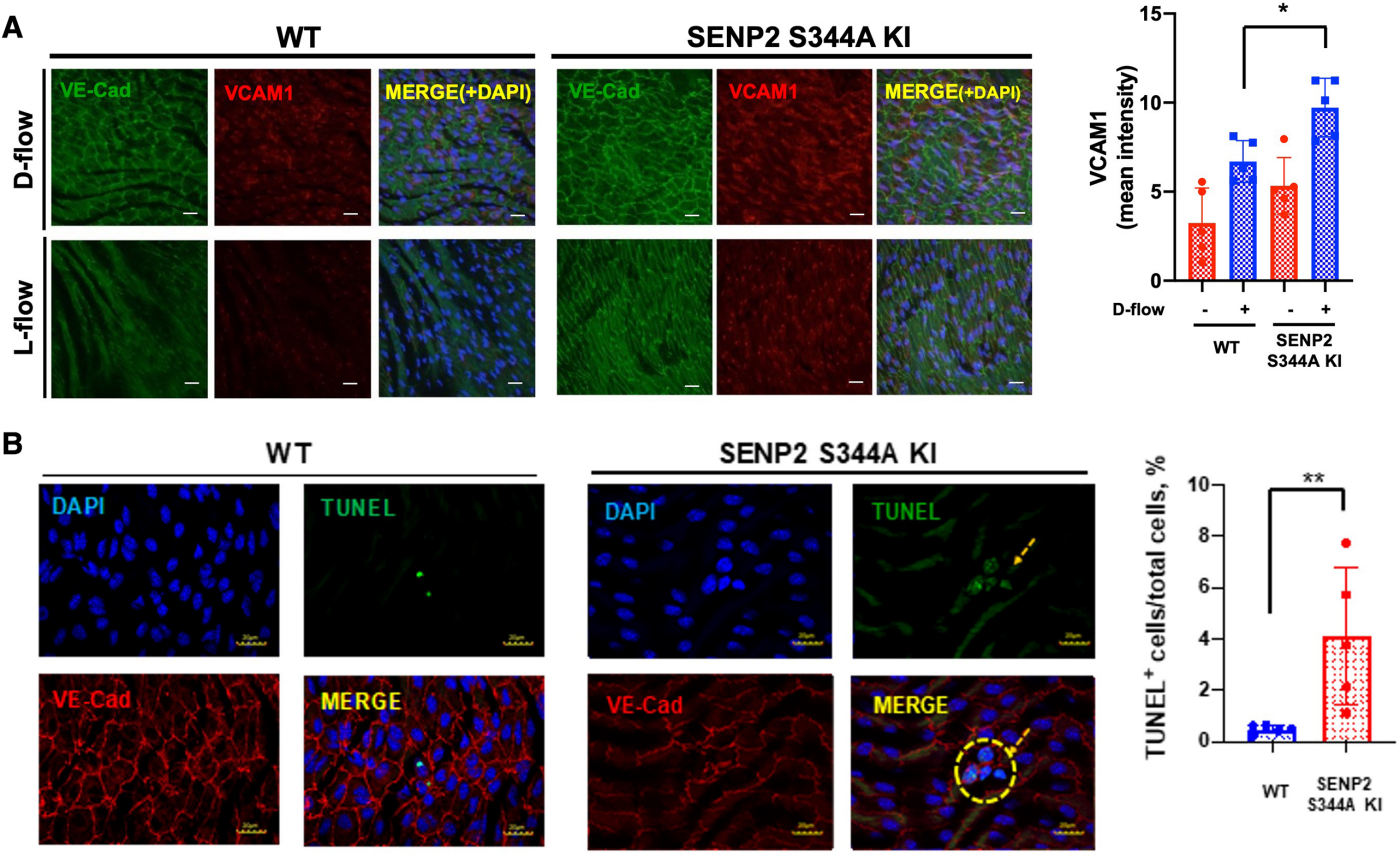
Increased EC inflammation and apoptosis in SENP2 S344A KI mice: (**A**) En face preparation of WT and SENP2 S344A KI aortas were immunofluorescence-stained with VCAM1 (red, EC inflammation) and VE-Cad (green, EC marker). (**B**) Aortic sections were stained with TUNEL (green, EC apoptosis (106)) and DAPI (blue, nucleus (9)). TUNEL-positive apoptotic nuclei were counted, indicating the colocalization between the TUNEL signal (representing fragmented DNA) and the DAPI signal (representing cell nuclei) (28). The graphs present quantified data from 5 independent samples (*n* = 5) (*p < 0.05, **p < 0.01, two-way ANOVA for (**A)**; unpaired *t*-test for (**B**)).

We have previously reported that SENP2 plays a crucial role in inhibiting EC inflammation and apoptosis by suppressing the SUMOylation of ERK5 and p53 (10, 57). While we did not observe an increase in SENP2 S344 phosphorylation in HUVECs exposed to D-flow *in vitro* (Figures 1B,D), our previous study has demonstrated that depletion of SENP2 leads to increased adhesion molecule expression and apoptosis in both L-flow and D-flow conditions in SENP2 knock-out mice (9). To further investigate the impact of SENP2 S344 phosphorylation on EC inflammation and apoptosis, we examined the effects in both L-flow and D-flow conditions using mice carrying the phospho-site mutation SENP2 S344A KI. Immunofluorescence staining revealed increased expression of VCAM1, a marker of EC inflammation, in the D-flow condition of the phospho-site mutation SENP2 S344A KI mice (Figure 2A). Additionally, TUNEL staining revealed an increased number of TUNEL-positive cells, indicating apoptosis, in the D-flow condition of the phospho-site mutation SENP2 S344A KI mice (Figure 2B). These findings provide further evidence supporting the role of SENP2 S344 phosphorylation in suppressing EC inflammation and apoptosis by preserving the deSUMOylation activity of SENP2 in an *in vivo* context.

### The phospho-site mutation SENP2 S344a KI mice exhibit larger lipid-laden lesions

**Figure 3:**
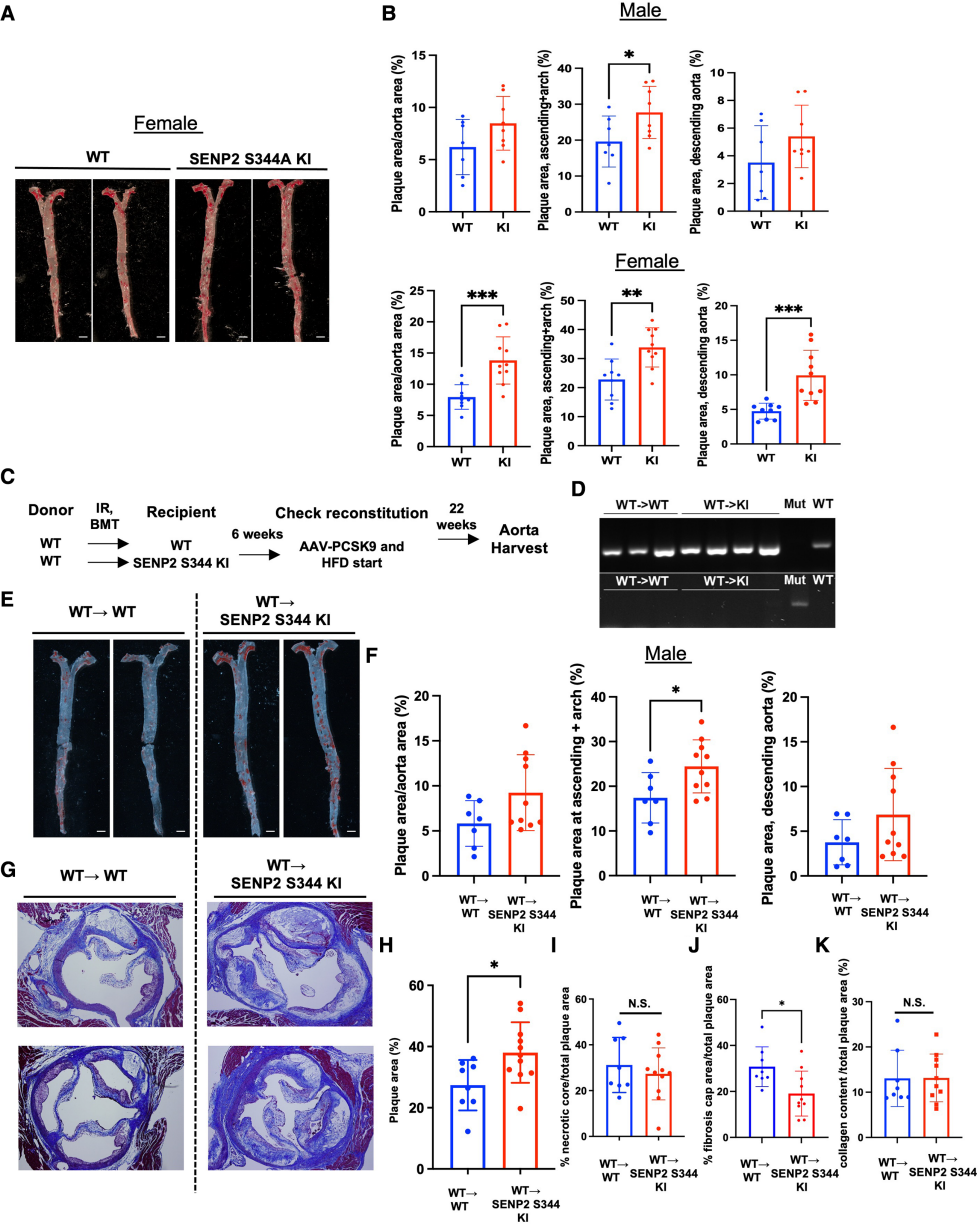
Increased lipid-laden lesions and fibrotic caps in SENP2 S344A KI mice: SENP2 S344A KI and WT mice were injected with a single dose of rAAV8-mPCSK9 and fed a high fat diet (HFD) for 16 weeks (42). (**A**) En face Oil Red O staining was performed to quantify lipid abundance and distribution. (**C**) The bone marrow transplantation (BMT) procedure was conducted (58). (**D**) Representative PCR data demonstrate the success of the BMT. (**E**) En face Oil Red O staining was performed on the BMT-generated models. (**G**) Aortic valve leaflet sections were stained with Masson's trichrome to evaluate changes in the aortic valves. (**B, F, H–K**) The graphs present quantified data from samples (*n* = 7–10) (*p < 0.05, **p < 0.01, unpaired *t*-test). N.S. indicates non-significant.

To assess the effect induced by SENP2 S344 phosphorylation in atherogenesis, we injected both WT control mice and phospho-site mutation SENP2 S344A KI mice with a single dose of rAAV8-mPCSK9 and fed them a high fat diet (HFD) for 16 weeks (42, 46). There were no differences in body weight and cholesterol levels between the WT and the phospho-site mutation SENP2 S344A KI mice (Supplementary Figures S1A,B). However, en face Oil Red O staining of mouse aortas showed significantly larger lipid-laden lesions in both the aortic arch (D--flow) and descending aorta (L-flow) of the female phospho-site mutation hypercholesteremic (HC) SENP2 S344A KI mice (Figures 3A,B, lower panel). In the male HC phospho-site mutation SENP2 S344A KI mice, significantly larger lipid-laden lesions were only observed in the aortic arch area (D-flow, Figure 3B, upper panel), suggesting that HC-mediated EC activation and atherogenesis are weaker in males compared to females. Because DNA damage-induced activation in cells of the vessel walls, i.e., ECs (14–16), is associated with atherogenesis, we determined whether the pro-atherosclerotic property of the phospho-site mutation SENP2 S344A KI is due to deficient SENP2 S344 phosphorylation in the cells of the vessel walls. Bone marrow cells in eight-to-nine-week-old SENP2 S344A KI or WT mice were ablated through lethal irradiation, then grafted with bone marrow cells extracted from WT donors of the same age (Figure 3C). Six weeks of recovery from bone marrow transplantation (BMT) (58), both WT → WT and WT → SENP2 S344A KI mice (Figure 3C) were injected with a single dose of rAAV8-mPCSK9 and fed a HFD (42) (Figure 3C). Twenty-two weeks after HFD, genomic DNA was extracted from peripheral blood and used for PCR to verify if BMT was successful (Figure 3D). There was no difference in body weight and cholesterol levels between WT→ WT and WT→SENP2 S344A KI (Supplementary Figures S1C,D) in both male and female mice. Although HC-mediated formation of lipid-laden lesions is weaker in systemic SENP2 S344A KI male mice compared to that of female mice, male WT→SENP2 S344A KI mice displayed larger lipid-laden lesions in D-flow areas (Figures 3E,F). Larger plaque lesions in the aortic valve cross-sectional areas were observed in WT → SENP2 S344A KI male mice compared to those in WT → WT male mice, suggesting that SENP2 S344 phosphorylation in the vessel wall plays a crucial role in inhibiting atherosclerotic formation, especially in D-flow areas (Figures 3G,H). Although the percentage of necrotic core formation per total lipid-laden areas was almost identical between male WT → SENP2 S344A KI and male WT → WT mice (Figure 3I), the fibrotic caps covering necrotic cores were thinner in male WT → SENP2 S344A KI (Figure 3J), suggesting that SENP2 S344 phosphorylation in cells of the vessels prevents vulnerable plaque formation (Figures 3I,J).

### Radiation accelerates EC activation through the downregulation of CHK1 expression

**Figure 4:**
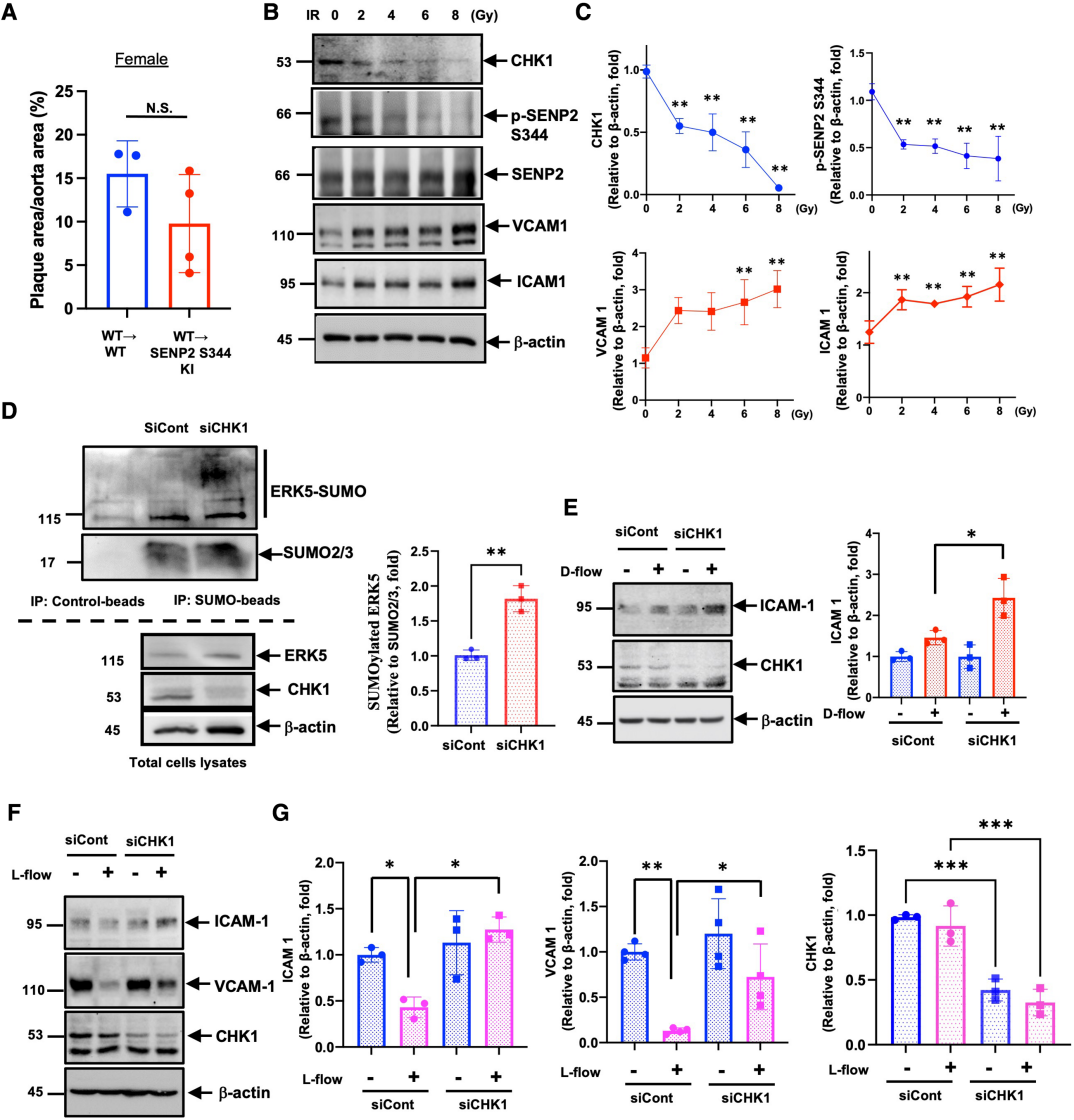
Radiation-induced EC activation through CHK1 downregulation: (**A**)there was no significant difference in lipid-laden lesions between female WT → WT and WT → SENP2 S344A KI mice (*n* = 3–4). (**B**) Expression levels of CHK1, SENP2 (p-S344 and total), VCAM1, ICAM1, and actin were evaluated in HUVECs exposed to 0, 2, 4, 6, 8 Gy of ionizing radiation (IR) for 24 h. (**C**) The graphs present quantified data from 3 independent experiments (**p < 0.01, two-way ANOVA). (**D**, left panel) SUMOylated ERK5 was assessed in siRNA-treated HUVECs, and the lysates were immuno-precipitated as described in Figure 1I. (**D**, right panel) The graphs present quantified data from 3 independent experiments (**p < 0.01, *t*-test). (**E**, left panel) siRNA-treated HUVECs were exposed to D-flow for 24 h, and the expression of ICAM1, CHK1, and actin were determined using immunoblotting. (**E**, right panel) The graphs present quantified data from 3 independent experiments (*p < 0.05, two-way ANOVA). (**F**) Expression levels of ICAM1, VCAM1, CHK1, and actin were evaluated in siRNA-treated HUVECs exposed to L-flow for 24 h using immunoblotting. (**G**) The graphs present quantified data from 3 or 4 independent experiments (*p < 0.05, **p < 0.01, two-way ANOVA).

Although en face Oil Red O staining showed that lipid-laden lesions in female SENP2 S344A KI mice were larger than in female WT mice (Figures 3A,B), when these female mice underwent BMT for vascular-specific models, the lipid-laden lesions in female WT → SENP2 S344A KI mice were almost identical to those in female WT → WT mice (Figure 4A). During BMT, mice were irradiated; therefore, we hypothesized that radiation may alter CHK1 and/or SENP2 activity. To address this possibilit*y*, we irradiated HUVECs and observed a dose-dependent reduction in both SENP2 S344 phosphorylation and CHK1 expression, accompanied by elevated expression of VCAM1 and ICAM1. The changes in VCAM1 and ICAM1 expression showed an inverse correlation with changes in CHK1 expression and SENP2 S344 phosphorylation (Figures 4B,C). Furthermore, CHK1 depletion accelerated ERK5 SUMOylation (Figure 4D) and D-flow-induced ICAM1 expression (Figure 4E), while counteracting the anti-inflammatory effect of L-flow (Figures 4F,G). These data suggest that radiation induces EC activation by suppressing the CHK1-SENP2 S344 phosphorylation axis. Therefore, the effect of the phospho-site mutation SENP2 S344A KI mice on atherosclerotic plaque formation became unclear, especially in female mice after BMT (Figure 4A), compared to the phospho-site mutation SENP2 S344A KI mice that did not receive radiation (Figures 3A,B lower panel).

### SENP2 S344 phosphorylation suppresses various processes contributing to EC activation

**Figure 5:**
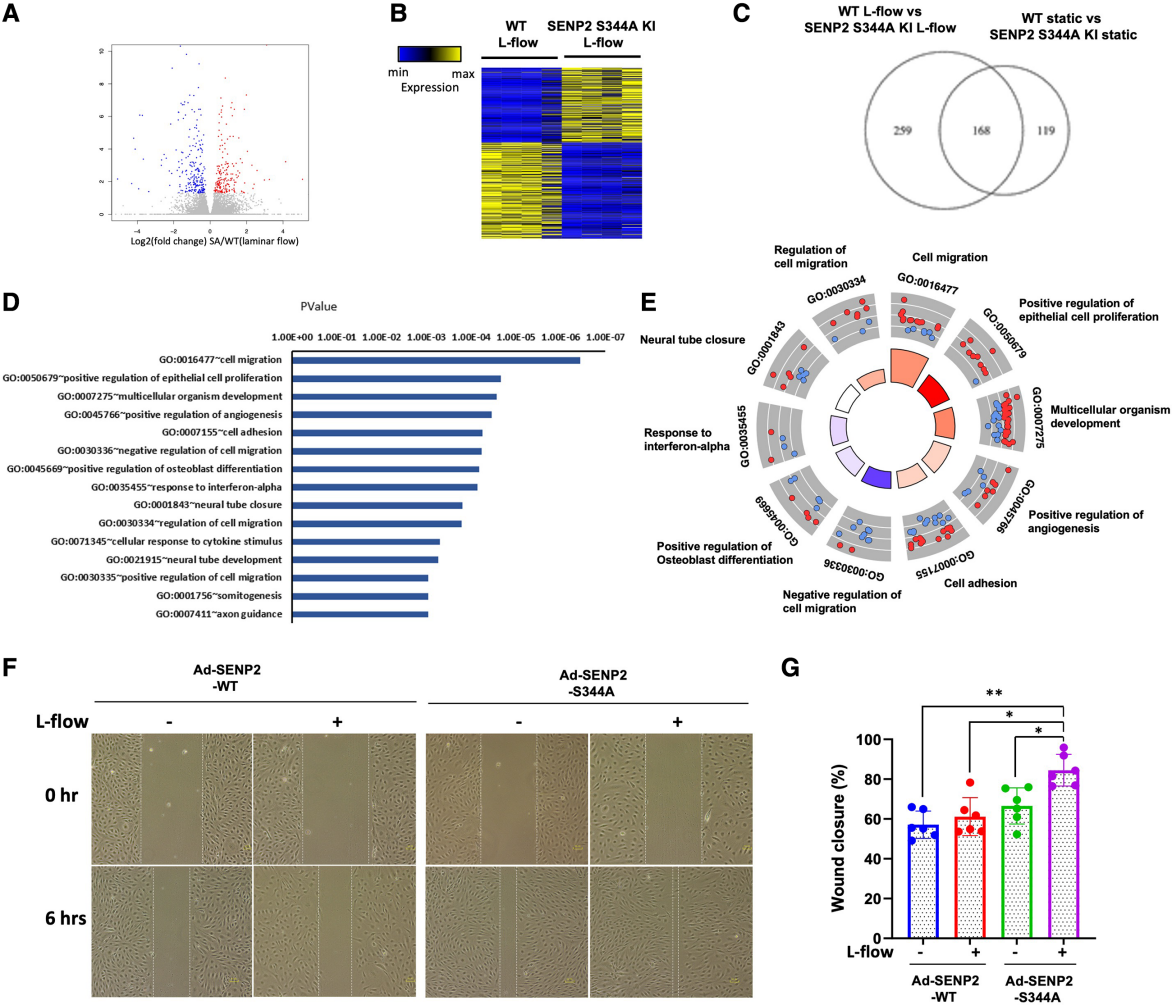
Increased EC pathophysiological stress response in SENP2 S344A KI MLECs: (**A**) the volcano plot illustrates the differentially expressed genes (DEGs) in WT MLECs compared to SENP2 S344A KI MLECs after L-flow; “red” dots represent upregulated DEGs, and “blue” dots represent downregulated DEGs. (**B**) A heatmap displaying hierarchical clustering of DEGs between WT MLECs vs. SENP2 S344A KI MLECs after L-flow for 24 h. (**C**) A Venn diagram indicating the total number of DEGs in WT MLECs and SENP2 S344A KI MLECs. (**D**) Annotation of EC activation-related gene ontology (GO) pathways identified in quadruplicate between WT MLECs vs. SENP2 S344A KI MLECs. (**E**) A molecular function Chord plot illustrating the enriched GO biological process terms associated with EC activation in SENP2 S344A KI MLEC transcripts, along with the corresponding genes arranged by their expression levels. (**F**) HUVECs transduced with Ad-SENP2 WT or Ad-SENP2 S344A were subjected to a scratch assay and exposed to L-flow for 6 h. Representative images of cell migration are shown. The dashed line represents the initial or final migration areas. (**G**) The graphs present quantified data from 6 independent experiments (*p < 0.05, **p < 0.01, two-way ANOVA).

To elucidate the functional role of L-flow-induced SENP2 S344 phosphorylation, SENP2 S344A KI MLECs and WT MLECs were exposed to L-flow for 24 h, and RNA-seq was performed. We identified 427 differentially expressed genes (DEGs) in SENP2 S344A KI MLECs compared to WT MLECs (Figure 5A). Among these DEGs, 259 out of 427 DEGs were regulated by L-flow (Figure 5B). GO analysis revealed that these 427 DEGs are associated with migration, positive regulation of epithelial cell proliferation, multicellular organism development, positive regulation of angiogenesis, and cell adhesion, all of which are known to contribute to EC activation (Figures 5D,E). GO Circle analysis revealed positive *Z*-scores (indicating activation) of all these processes in SENP2 S344A KI MLECs (Figure 5E). Furthermore, an *in vitro* study showed that L-flow promotes the migration of HUVECs, and this effect was further enhanced by the transduction of Ad-SENP2 S344A phospho-site mutation (Figures 5F,G). Activation of ERK5 transcriptional activity can suppress EC migration (59), and our previous study has demonstrated that ERK5 SUMOylation can inhibit ERK5 transcriptional activity (49). Therefore, the increased migration of HUVECs driven by the transduction of Ad-SENP2 S344A can be attributed to an increase in ERK5 SUMOylation. Collectively, our findings suggest the involvement of SENP2 S344 phosphorylation in regulating various processes contributing to EC activation, including EC migration.

### SENP2 S344 phosphorylation suppresses fibrotic changes concurrent with upregulating EC-specific gene expression

**Figure 6:**
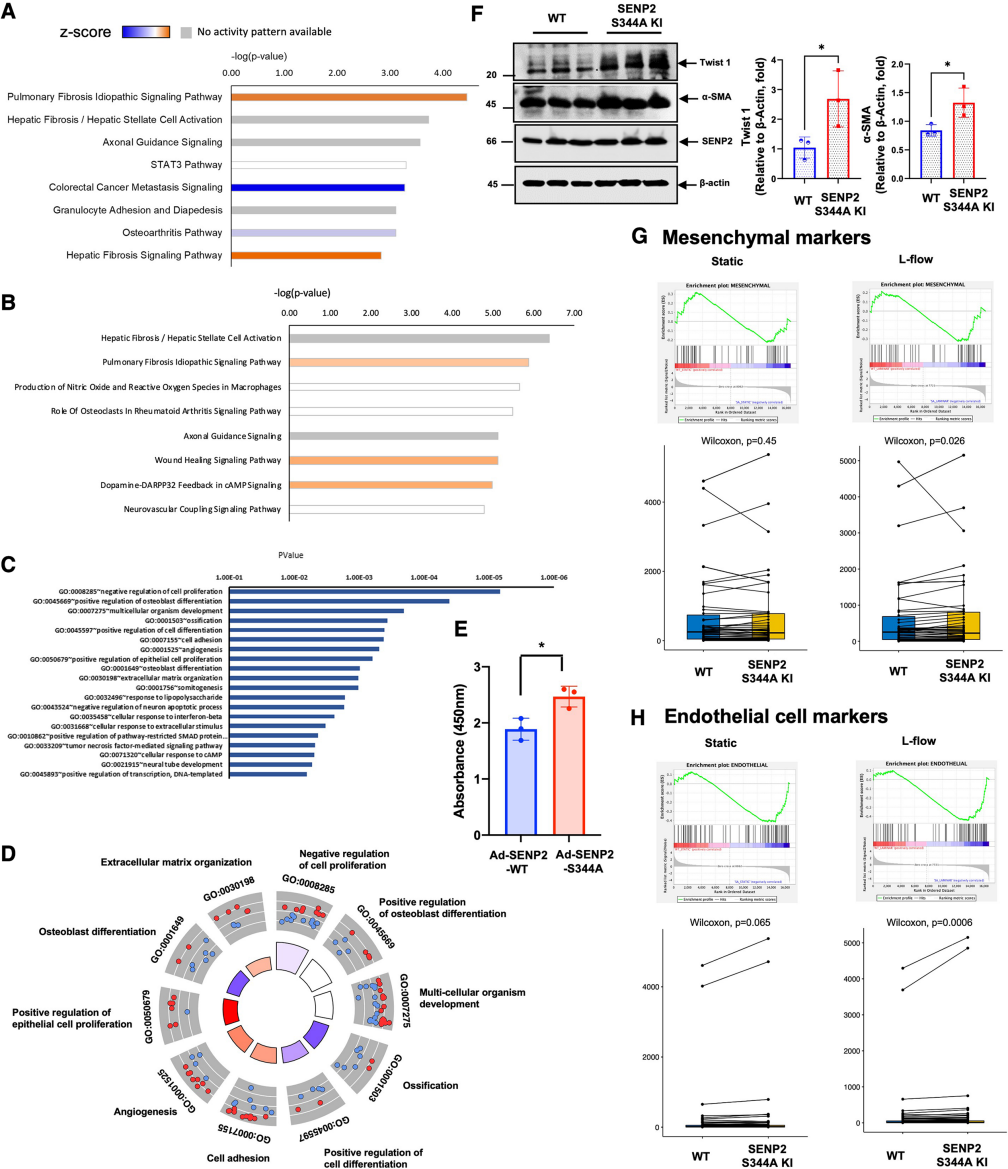
Increased EC activation-stimulated fibrosis in SENP2 S344A KI MLECs with upregulated expression of EC markers: (**A**) after L-flow, the *Z*-score of fibrosis-related processes is positively upregulated in SENP2 S344A KI MLECs, as determined by IPA analysis. (**B**) Under static condition, the *Z*-score of the pulmonary fibrosis idiopathic signaling pathway remains positive in SENP2 S344A KI MLECs. (**C**,**D**) Under static condition, the *Z*-score of EC activation-associated processes, including cell adhesion, angiogenesis, positive regulation of epithelial cell proliferation, and extracelular matrix organization, is positive in SENP2 S344A KI MLECs. (**E**) DNA synthesis is increased in HUVECs transduced with Ad-SENP2 S344A compared to HUVECs transduced with Ad-SENP2 WT, as demonstrated by data from 3 independent experiments. (**F**, left panel) The expression of mesenchymal markers (TWIST1, αSMA) is increased in SENP2 S344A KI MLECs. (**F**, right panel) The graphs present quantified data from 3 independent experiments (*p < 0.05, *t*-test). (**G**,**H**) The expression of both mesenchymal (**G**) and endothelial (**H**) markers is increased in SENP2 S344A KI MLECs.

Based on the knowledge-based database of Ingenuity Pathways Analysis (IPA), we identified the pulmonary fibrosis idiopathic signaling pathway as the top-ranked enriched IPA canonical pathway. Moreover, the *Z*-score (a measurement of activation) from IPA suggests that fibrotic changes-related processes in the pulmonary fibrosis idiopathic signaling pathway and hepatic fibrosis signaling pathway are positively upregulated in SENP2 S344A KI MLECs compared to WT MLECs after L-flow (Figure 6A; Supplementary Table S1). Even in static conditions, the *Z*-score of the pulmonary fibrosis idiopathic signaling pathway remains positive, further suggesting the pro-fibrotic effects of the phospho-site mutation SENP2 S344A KI MLECs (Figure 6B). Importantly, the *Z*-score of all processes contributing to EC activation, such as cell adhesion, angiogenesis, positive regulation of epithelial cell proliferation, and extracellular matrix organization, is also positive in SENP2 S344A KI MLECs under static conditions. These data strongly suggest the contribution of SENP2 S344 phosphorylation to EC activation (Figures 6C,D). Consistent with these findings, we observed an increase in DNA synthesis in SENP2 S344A KI MLECs compared to WT MLECs (Figure 6E). To evaluate the role of SENP2 S344 phosphorylation in fibrotic changes, we determined the expression levels of fibrotic and mesenchymal markers and found that both TWIST1 (fibrotic marker) and αSMA (mesenchymal marker) expression were increased in SENP2 S344A KI MLECs (Figure 6F). Since d-flow can activate endoMT, we also assessed the expression of both mesenchymal and EC-specific genes. We found that the expression of both mesenchymal and EC-specific gene was upregulated in SENP2 S344A KI MLECs after L-flow (Figures 6G,H). These data suggest that the phospho-site mutation SENP2 S344A KI MLECs exhibit a distinct expression pattern, promoting fibrotic changes while maintaining EC phenotypes without inducing endoMT. This distinct expression pattern may account for the decrease in fibrotic cap area without changing collagen composition in phospho-site mutation SENP2 S344A KI mice (Figure 3K) because a decrease in fibrotic cap areas did not cause a parallel decrease in collagen composition.

### Via SENP2 S344 phosphorylation and ERK5 activation, L-flow upregulates the expression of DDIAS, which inhibits EC apoptosis

**Figure 7:**
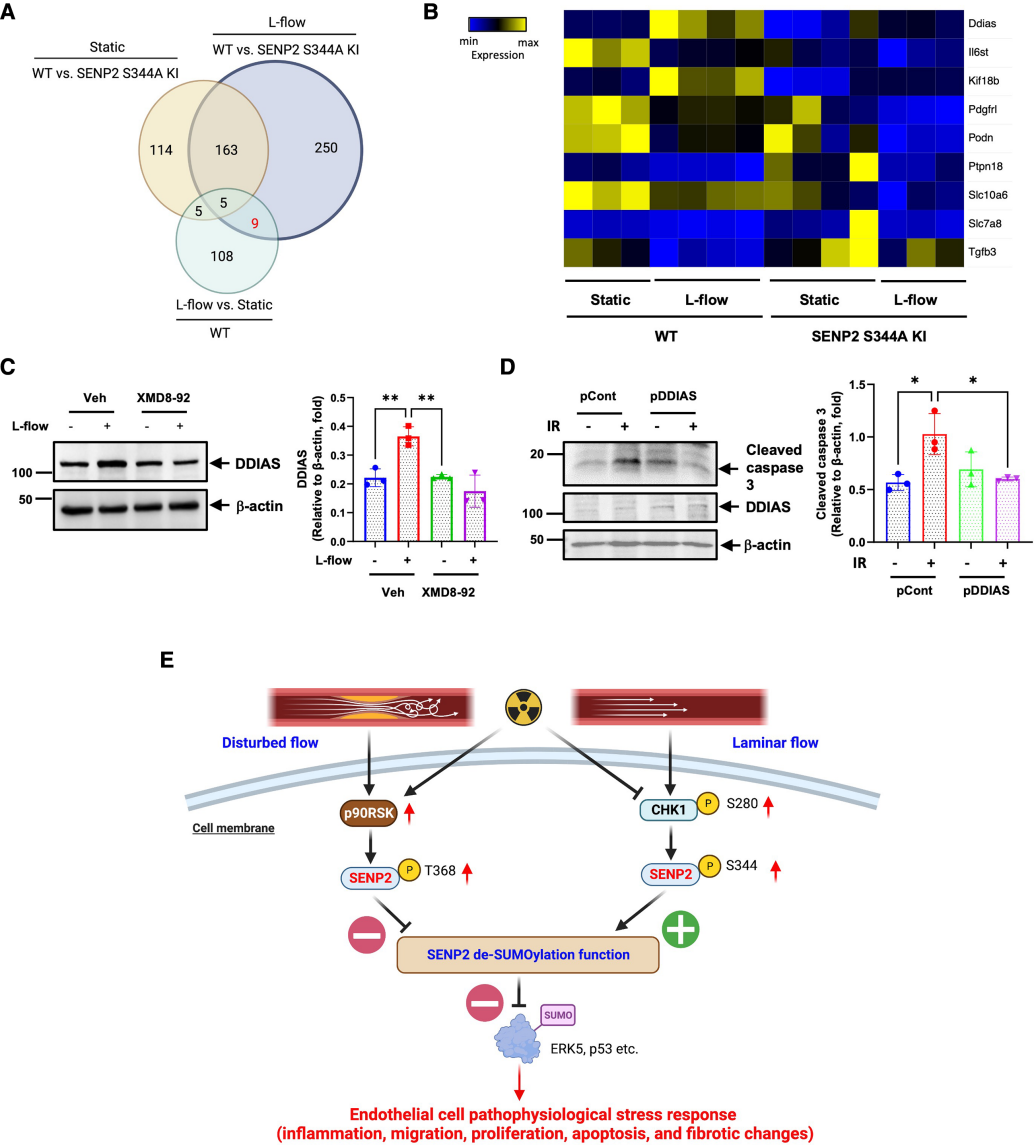
L-flow upregulates DDIAS expression via SENP2 S344 phosphorylation and ERK5 activation to suppress EC apoptosis: (**A**) the venn diagram illustrates the significant DEGs between the groups. (**B**) The heatmap displays the nine core genes, including DDIAS. (**C**, left panel) Pretreatment with XMD8-92 (5 µM), an ERK5-specific inhibitor, suppresses L-flow-mediated upregulation of DDIAS expression in HUVECs after 24 h. (**C**, right panel) The graphs present quantified data from 3 independent experiments (**p < 0.01, two-way ANOVA). (**D**, left panel) HUVECs were overexpressed with DDIAS using the DDIAS plasmid or control plasmid, then exposed to IR for 24 h. The increased expression of cleaved caspase-3 induced by IR is reversed by DDIAS overexpression. (**D**, right panel) The graphs present quantified data from 3 independent experiments (*p < 0.05, two-way ANOVA). “pCont” refers to the control plasmid, and “pDDIAS” refers to the DDIAS plasmid. (**E**) The scheme illustrates the mechanism of L-flow-induced SENP2 S344 phosphorylation in suppressing the EC pathophysiological stress response.

Venn diagram analysis revealed 117 DEGs in the WT MLECs after L-flow. Among these DEGs, 14 out of 117 DEGs were regulated by SENP2 S344 phosphorylation (Figure 7A). Notably, *Ddias*, *II6st*, *Kif18b*, *Pdgfrl*, *Podn*, *Ptpn18*, *Stc10a6*, *Slc7ab*, and *Tgfb3* were identified as 9 core genes regulated by L-flow-induced SENP2 S344 phosphorylation (Figures 7A,B). These 9 core genes likely play a critical role in various processes contributing to EC activation, as depicted in Figures 6C,D. Interestingly, we observed that the expression of transforming growth factor beta 3 (*Tgfb3*), a regulator of fibrotic changes (60), was inhibited by L-flow, and this inhibition was abolished in the SENP2 S344A KI MLECs (Figure 7B). These findings suggest that Tgfb3 is involved in the anti-fibrotic effects mediated by SENP2 S344 phosphorylation. Furthermore, a heatmap analysis of these 9 core genes revealed that *Ddias* was one of the most significantly upregulated genes (Figure 7B). As *Ddias* expression is regulated by ERK5 activation and has been shown to inhibit cell death (61, 62), we investigated whether L-flow-induced ERK5 activation regulates DDIAS expression. In HUVECs, we found that the upregulated expression of DDIAS induced by L-flow was abolished when treated with XMD8-92, an ERK5 specific inhibitor (Figure 7C). Previously, we reported that ERK5 transcriptional activity is involved in ionizing radiation (IR)-induced EC apoptosis (63). To assess the role of DDIAS expression in EC apoptosis, we exposed HUVECs expressing DDIAS to 2 Gy of IR and observed that the increase in cleaved caspase 3 expression induced by IR was attenuated (Figure 7D). These findings collectively highlight the crucial role of DDIAS in the anti-apoptotic effects mediated by L-flow.

## Discussion

CHK1 phosphorylates SENP2 at S344 (19), which was discovered as a new residue through mass spectrometry-based phospho-proteomics but with uncharacterized functions (38, 39). In this study, we present evidence describing the functional role and regulatory mechanism governing SENP2 S344 phosphorylation in ECs after L-flow. L-flow enhances the phosphorylation of CHK1 S280 and SENP2 S344, leading to an increase in SENP2 deSUMOylation activity. This is supported by the suppression of ERK5 and p53 SUMOylation and the inhibition of EC inflammation and apoptosis. The phospho-site mutation SENP2 S344A KI mice exhibit an increase in adhesion molecule expression and apoptosis, as well as larger lipid-laden lesions in both female and male mice undergoing HC-mediated atherosclerosis compared to the WT mice, although HC-mediated atherogenesis is weaker in male mice. However, when these mice undergo BMT, lipid-laden lesions in the female WT → SENP2 S344A KI mice are almost identical to those of the female WT → WT mice. We found that radiation can alter the anti-atherosclerotic effect of SENP2 S344 phosphorylation by downregulating the expression of CHK1*.* Therefore, interpreting the anti-atherosclerotic effect of CHK1-SENP2 S344 phosphorylation in the model of atherosclerosis after BMT would be challenging. Importantly, we found that the CHK1-SENP2 S344 phosphorylation axis coordinately suppresses multiple processes associated with fibrotic changes concurrent with EC activation, such as inflammation, migration, proliferation, and apoptosis. The deficient SENP2 S344 phosphorylation instigates various processes associated with fibrotic changes, along with upregulated expression of EC-specific genes, suggesting that L-flow-induced CHK1-SENP2 S344 phosphorylation promotes fibrotic changes through a mechanism distinct from endoMT. Lastly, we found that by activating ERK5, L-flow upregulates the expression of DDIAS, which subsequently suppresses EC apoptosis by phosphorylating SENP2 S344. Taken together, our data suggest the key role of the CHK1-SENP2 S344 phosphorylation axis in regulating various processes contributing to both EC activation and fibrotic changes in concert. This process is initiated by DDR (CHK1) activation and inhibits both EC activation and fibrotic changes by inducing SENP2 deSUMOylation activity. Our findings suggest that the CHK1-SENP2 S344 phosphorylation axis may suppress excessive EC activation and fibrotic changes after various pathophysiological stresses for maintaining EC barrier function, especially under L-flow.

In this study, we found that L-flow induces the CHK1-SENP2 S344 phosphorylation axis, which inhibits EC inflammation (including adhesion molecule expression), migration, proliferation, apoptosis, and fibrotic changes. These components are critical in the EC response to pathophysiological stress. It is well known that L-flow inhibits these processes, while D-flow activates them (20). Our findings demonstrate that L-flow-induced CHK1-SENP2 S344 phosphorylation increases SENP2 deSUMOylation activity, leading to the inhibition of ERK5 and p53 SUMOylation. In contrast, our previous reports have shown that D-flow-induced p90RSK-SENP2 T368 phosphorylation inhibits SENP2 function in the nucleus, resulting in increased SUMOylation of ERK5 and p53 (9). Together, these findings highlight the crucial role of SENP2 deSUMOylation activity in regulating various processes associated with the EC response to pathophysiological stress, including inflammation, migration, proliferation, apoptosis, and fibrotic changes in concert. These processes occur under two different flow patterns, utilizing two different SENP2 phosphorylation sites: T368 by p90RSK and S344 by CHK1. These phosphorylation sites play a vital role in determining SENP2 deSUMOylation function and subsequent pro- vs. anti-EC activation (Figure 7E). Furthermore, we have previously reported that IR increases p90RSK activation, which phosphorylates SENP2 T368 (63, 64). Therefore, through its dual effects of downregulating CHK1 expression and activating p90RSK, IR can induce a severe EC response to pathophysiological stress, especially under flow conditions *in vivo* (Figure 7E). Nevertheless, further studies are necessary to clarify these possibilities.

ECs are exposed to various stimuli, including blood flow, inflammation, and metabolites, which can influence their phenotypes and functions. While the plasticity and heterogeneity of ECs can allow them to adapt to these alterations (65), certain stimuli can lead to diseases. For instance, inflammation accompanied by increased TGFβ expression can trigger reactivation of endoMT (65, 66), resulting in fibrotic disorders and cardiac fibrosis (29, 67–72). Additionally, D-flow can activate endoMT, contributing to the development of atherosclerosis (31, 73–75). Our RNA-seq analysis revealed that SENP2 S344 phosphorylation plays a negatively regulatory role in pulmonary fibrosis idiopathic signaling and hepatic fibrosis/hepatic stella cell activation pathways, suggesting its involvement in regulating fibrotic changes. However, unlike endoMT, we observed an upregulation of both mesenchymal and EC-specific markers in SENP2 S344A KI MLECs compared to WT MLECs after L-flow. This pattern has also been reported in ECs of glaucomatous Schlemm's canal (76). We propose that the fibrotic changes observed in SENP2 S344 KI MLECs, without overt endoMT phenotypes, represent a unique type of EC response to pathophysiological stress.

The role of DDR in blood flow-regulated EC function is not yet established. ATR, which responds to single-stranded DNA breaks by activating the DNA damage checkpoint through phosphorylation of CHK1 S345, can be activated by mechanosensitive ion channel Piezo and NO signaling independently of DNA damage (77). In ous study, we observed that neither L-flow nor d-flow increased CHK1 S345 phosphorylation. However, L-flow did increase CHK1 S280 phosphorylation, which facilitates CHK1 translocation from the cytoplasm to the nucleus and activates downstream CHK1 signaling in response to serum stimulation (55). These findings suggest that ATR activation may not be involved in CHK1 function. Since L-flow induced SENP2 S344 phosphorylation without affecting CHK1 S345 phosphorylation, it is possible that L-flow-induced CHK1 nuclear translocation plays a crucial role in promoting nuclear SENP2 S344 phosphorylation. The mechanism underlying L-flow-induced CHK1 S280 phosphorylation remains unknown and requires further investigation.

We made an unexpected observation that radiation downregulates CHK1 expression in ECs. To the best of our knowledge, this is the first study to demonstrate a reduction in CHK1 protein expression following radiation exposure. While it is known that p53 and RB1/E2F1 can upregulate CHK1 promoter activity (78) and CHK1 ubiquitination has been reported (79), the specific mechanism by which radiation inhibits CHK1 expression in ECs remains unclear. Given that we have established the role of CHK1-mediated SENP2 S344 phosphorylation in EC fibrotic changes, but not in endoMT, it is possible that radiation induces EC fibrotic changes without triggering endoMT, thereby contributing to the formation of vulnerable plaques. Although radiation-induced endoMT associated with radioresistance in cancer has been reported (80), the role of EC fibrotic changes in radioresistance has not been explored. Therefore, it is crucial to investigate the CHK1-SENP2 S344 phosphorylation axis to understand radiation-induced cardiovascular events and resistance against tumorigenesis. Our findings shed light on a novel aspect of CHK1 biology and its potential implications in how blood flow influences atherogenesis. This knowledge informs current preclinical and clinical interest in CHK1 inhibitors.

While epidemiological and experimental studies have demonstrated the detrimental cardiovascular effects of high doses of IR, the impact of low-dose IR remains unclear (81, 82). A study by Schöllnberger et al. (83, 84) did not definitively establish a risk of heart disease for individuals exposed to doses below 2.6 Gy, while Azizova et al. (85) indicated an increased risk of developing ischemic heart disease for cumulative external doses above 1 Gy. However, there is a lack of epidemiological data on the impact of radiation on cardiovascular diseases after exposure to low to moderate doses, particularly below 500 mGy (86–93). Several experimental studies have focused on investigating the effects of low-dose radiation using mouse models of atherosclerosis, specifically genetically modified ApoE knocked out (KO) mice fed a chow diet. These studies underscore the importance of considering the dose-rate of radiation exposure (94). Mitchel et al. (95, 96) demonstrated that exposure to low-dose IR, especially at a low dose rate, slowed plaque progression in mice. Conversely, Mancuso et al. (97) indicated that acute irradiation at moderate doses (300 mGy) can have detrimental effects on atherosclerosis, whereas chronic exposure to the same dose has a lesser impact. Additionally, both Le Gallic et al. (98) and Ebrahimian et al. (99) found that chronic internal low-dose IR enhances plaque stability in ApoE KO mice. Furthermore, these studies (98, 99) showed a decrease in inflammatory parameters following exposure to low-dose IR, such as reduced plaque content of CD68+ foam cells and a shift in aortic mRNA expression favoring anti-inflammatory cytokines over pro-inflammatory ones (98, 99). These findings support the notion that IR affects the immune system, with high doses promoting inflammation (100) and pro-inflammatory macrophages (101), while lower doses lead to decreased inflammation (102). In a study by Rey et al., the immunomodulatory response to different doses of low-dose IR was investigated. The study utilized ApoE KO mice and examined cumulative doses ranging from 50 to 1,000 mGy, with dose-rates of γ rays set at very low (1.4 mGy h^−1^) or low (50 mGy h^−1^) levels. The results revealed a significant decrease in pro-inflammatory Ly6CHi monocytes at all cumulative doses when exposed to low dose-rate radiation. However, at very low dose-rates, reductions in Ly6CHi cells were observed only at doses of 50, 100, and 750 mGy. Conversely, the proportions of Ly6CLo monocytes were not affected by low-dose IR. Additionally, the proportions of CD4+ T cell subsets in the spleen showed no differences between irradiated mice and non-irradiated controls, whether assessing CD25+FoxP3+ regulatory or CD69+ activated lymphocytes. Within the aorta, the gene expression of cytokines such as IL-1 and TGF-β, as well as adhesion molecules including E-Selectin, ICAM-1, and VCAM-1, were reduced at an intermediate dose of 200 mGy. These findings suggest that low-dose IR may decrease the formation of atherosclerotic plaques by selectively reducing pro-inflammatory monocytes in the bloodstream and impairing adhesion molecule expression and inflammatory processes in the vessel wall. In contrast, the splenic T lymphocytes showed no significant effects from low-dose IR. Notably, some responses to irradiation exhibited nonlinear behavior, as reductions in aortic gene expression were significant at intermediate doses but not at the highest or lowest doses. This study contributes to our understanding of how low-dose IR with different dose-rates impacts the immune system response in the context of atherosclerosis (103). Furthermore, in smooth muscle cell lineage-tracing mice (Myh11-ERT2Cre ROSA-STOP-eYFP) on the ApoE KO background, total body γ-irradiation of 12 Gy and subsequent bone marrow reconstitution led to the loss of smooth muscle cell investment in lesions induced by an 18-week Western diet. This effect was observed in the brachiocephalic artery, carotid arteries, and the aortic arch, but not in the aortic root or abdominal aorta (104). Ikeda et al. reported that IR has a suppressive effect on the initiation of atherogenesis in the aortic arch by inhibiting the accumulation of LDL in the intima. However, as time progresses, the growth of lesions is influenced by delayed accumulation of neutral lipid and myeloid cells in the intima, as well as reduced coalescence of small foam cell clusters into larger lesions. Notably, the lateral expansion of lesions, as measured by the lesion outline area, appears to be minimally affected. This suggests that the lateral expansion of lesions may primarily depend on d-flow or other factors that are not influenced by irradiation. Additionally, other mechanisms may contribute to the phenotype observed after BMT, particularly at later stages when the height of lesions is significantly reduced. Single-cell transcriptomic analysis indicates that IR diverts LDL uptake by ECs towards lysosomal degradation and reverse cholesterol transport pathways. This diversion leads to a reduction in intimal lipid accumulation and impacts the initiation and growth of lesions without affecting paracellular leakage (105). These findings demonstrate the complexity of the relationship between radiation and atherosclerosis, which can depend on various factors, including the dose and duration of radiation exposure, the specific tissues or organs affected, and individual susceptibility. While some studies suggest that radiation exposure may contribute to the development or progression of atherosclerosis, other research findings are inconclusive or even indicate potential protective effects. It is important to acknowledge that radiation is employed in medical treatments like radiation therapy, which can have both therapeutic benefits and potential side effects. The impact of radiation on atherosclerosis may vary depending on the context in which it is applied, such as therapeutic radiation versus accidental exposure. Therefore, further research is required to gain a comprehensive understanding of the association between radiation and atherosclerosis. However, the findings from the study by Ikeda et al. provide intriguing insights. According to their research, the lesions observed in our BMT model appear to rely on hemodynamics rather than radiation (105). These findings align perfectly with the objectives of our own study.

We observed a downregulation of CHK1 protein expression in ECs following radiation exposure; however, the specific mechanisms involved remain unclear and require further research. Our findings suggest a potential association between radiation-induced EC fibrotic changes and vulnerable plaque formation, but a comprehensive investigation of the relationship between radiation, endoMT, and atherosclerosis is still needed. Furthermore, it is important to acknowledge that *in vitro* experiments and animal models may not fully capture the intricacies of human physiology and disease. Hence, additional studies utilizing human samples and clinical research are necessary to validate these findings and determine their relevance to human cardiovascular health. Moreover, it should be noted that this study does not focus on the clinical implications or long-term outcomes of radiation-induced cardiovascular events or resistance against tumorigenesis, as they lie beyond the scope of our research. Consequently, future investigations should aim to address these aspects and provide a more comprehensive understanding of the potential impact of the CHK1-SENP2 S344 phosphorylation axis and radiation exposure on human health.

## Data Availability

The datasets presented in this study can be found in online repositories. The names of the repository/repositories and accession number(s) can be found below: https://www.ncbi.nlm.nih.gov/genbank/, GSE222511.

## Supplementary material

SUPPLEMENTARY FIGURE S1

(**A,C**) Body weight and (**B,D**) cholesterol levels in HC-induced (**A,B**) and BMT (**C,D**) atherosclerosis. Mice with LDL cholesterol level <250 mg/dl were excluded from the HC-induced atherosclerosis analysis (**B**), and mice with LDL cholesterol level <200 mg/dl were excluded from the BMT atherosclerosis analysis (**D**). N.S, non-significant.
