# Supplementary figures and images for: Endothelial activation and fibrotic changes are impeded by laminar flow-induced CHK1-SENP2 activity through mechanisms distinct from endothelial-to-mesenchymal cell transition
Source: Front Cardiovasc Med. 2023 Aug 30;10:1187490. doi: 10.3389/fcvm.2023.1187490 (PMC10499395; doi:10.3389/fcvm.2023.1187490)

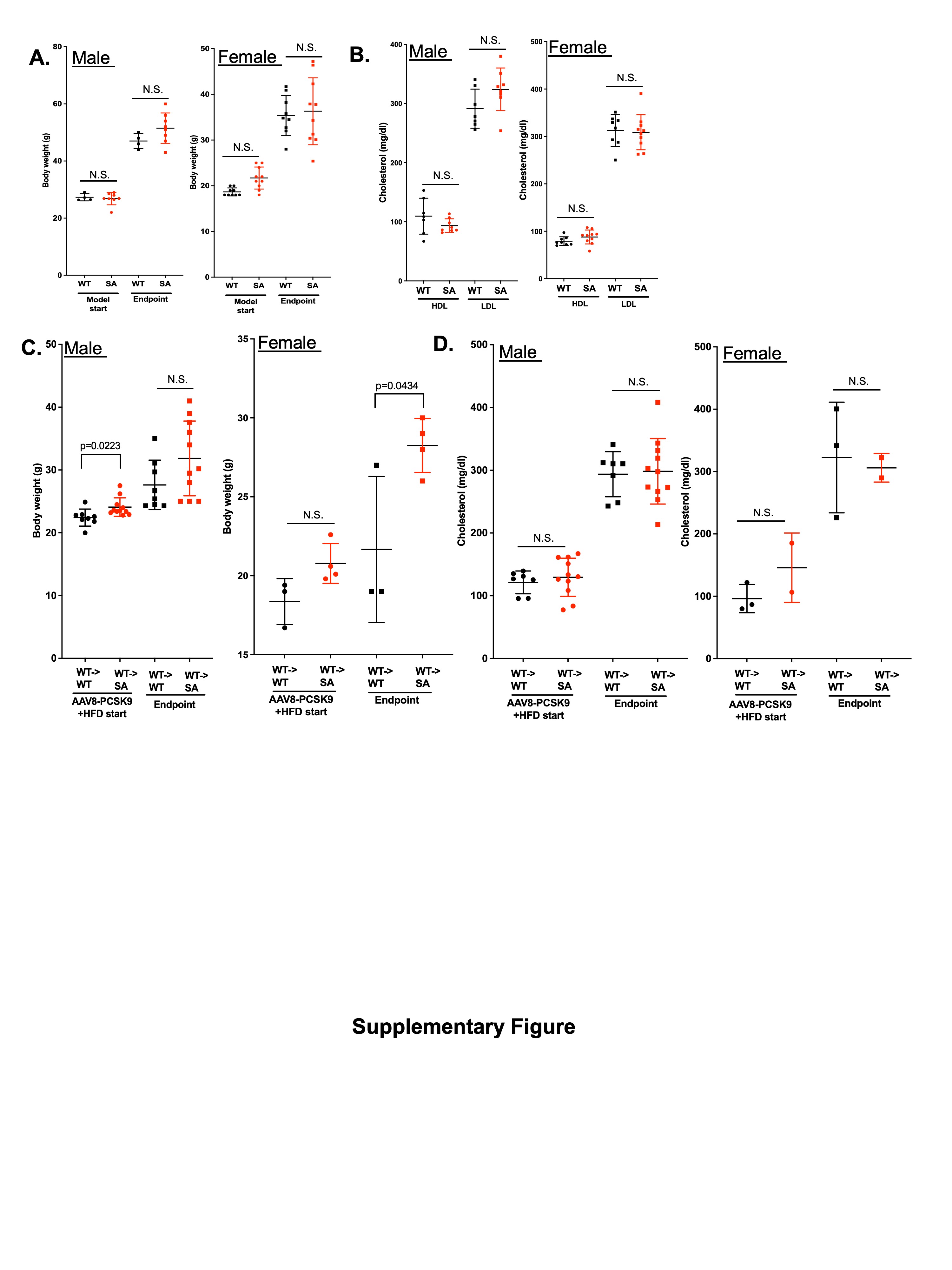

Supplement: Supplementary file 1 [file Image1.jpeg]

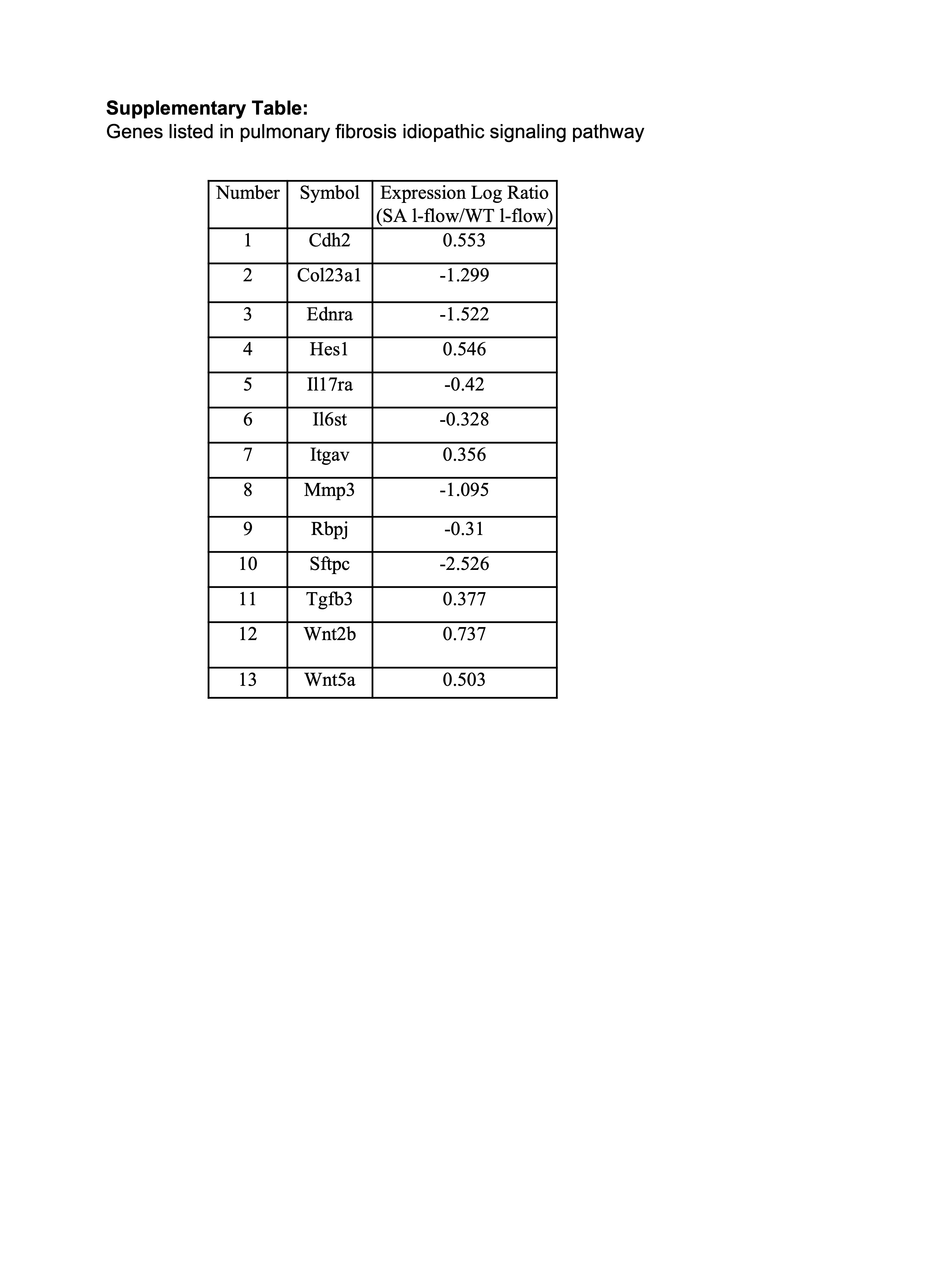

Supplement: Supplementary file 2 [file Image2.jpeg]
